# A Plant-Produced Bacteriophage Tailspike Protein for the Control of *Salmonella*

**DOI:** 10.3389/fpls.2015.01221

**Published:** 2016-01-08

**Authors:** Sean Miletic, David J. Simpson, Christine M. Szymanski, Michael K. Deyholos, Rima Menassa

**Affiliations:** ^1^Southern Crop Protection and Food Research Centre, Agriculture and Agri-Food Canada, LondonON, Canada; ^2^Department of Biology, University of Western Ontario, LondonON, Canada; ^3^Alberta Glycomics Centre and Department of Biological Sciences, University of Alberta, EdmontonAB, Canada; ^4^Department of Biology, University of British Columbia, KelownaBC, Canada

**Keywords:** bacteriophage tailspike protein, chickens, *Nicotiana benthamiana*, plant biotechnology, *Salmonella*, transient transformation

## Abstract

The receptor binding domain of the tailspike protein Gp9 from the P22 bacteriophage was recently shown to reduce *Salmonella* colonization in the chicken gut. In this study, we transiently expressed the receptor binding domain of the Gp9 tailspike protein in *Nicotiana benthamiana*, and targeted it to the endoplasmic reticulum (ER) or to the chloroplasts. Gp9 was also fused to either an elastin-like polypeptide (ELP) or hydrophobin I tag, which were previously described to improve accumulation levels of recombinant proteins. The highest levels of recombinant protein accumulation occurred when unfused Gp9 was targeted to the ER. Lower levels of chloroplast-targeted Gp9 were also detected. ELP-fused Gp9 was purified and demonstrated to bind to *Salmonella enterica* serovar Typhimurium *in vitro*. Upon oral administration of lyophilized leaves expressing Gp9-ELP to newly hatched chickens, we found that this tailspike protein has the potential to be used as a therapeutic to control *Salmonella* contamination in chickens.

## Introduction

The human intestine is a complex microbial ecosystem where hundreds of species of bacteria have adapted to live and grow. A mutualistic relationship has evolved benefiting the health of the host while providing an optimal habitat for microflora to thrive (Guarner and Malagelada, 2003). However, some of these bacteria are pathogenic, causing a wide array of intestinal pathologies. *Salmonella enterica* is a Gram-negative enteropathogenic bacterium that is widely prevalent and is one of the primary causes of foodborne illness in humans. There are roughly 1.4 million non-typhoidal salmonellosis cases each year in North America, causing approximately 25% of all hospitalizations due to foodborne illness (Mead et al., 1999). *S. enterica* serotype Typhimurium also referred to as *S.* Typhimurium, causes gastroenteritis characterized by diarrhea, vomiting, and abdominal pain and is showing the emergence of multidrug-resistant strains (Su et al., 2004; Chen et al., 2013). Poultry and eggs are a major source of infection, but other sources such as vegetables, fruits, nuts, sprouts, leafy greens, roots, and beans have been reported (Rodrigue et al., 1990; Hammack, 2012). In chickens, *Salmonella* is found throughout the intestinal tract (Fanelli et al., 1971) and the rupturing of intestinal contents during evisceration can readily contaminate poultry meat. For instance, *Salmonella* has been isolated from 33% of raw chicken breasts sampled from retail grocery stores in Ontario, Canada (Cook et al., 2012).

Antibiotic use has led to the emergence of antibiotic-resistant *Salmonella* strains. In 2013, 17% of typhoidal *Salmonella* isolates from Canadians were resistant to ciprofloxacin and 41% of *S.* Heidelberg infections were resistant to at least one antibiotic^1^. This growing concern has provoked research into alternative methods for controlling bacterial outbreaks. Considerable research into using bacteriophage therapy to treat or prevent bacterial infections progressed in Eastern Europe and the former Soviet Union during the latter part of the 20th century and could potentially be reconsidered as a viable alternative to antibiotics (Sulakvelidze et al., 2001). Lytic bacteriophages are host-specific, self-replicating, and virtually non-toxic making them attractive alternatives to control bacteria such as *Salmonella* and bacteriophages have been shown to reduce *Salmonella* colonization in chickens (Goode et al., 2003; Atterbury et al., 2007). Despite these successes, this therapy is not without drawbacks. Bacteriophages are host-specific requiring diagnosis of the pathogen before the phage is administered (Waseh et al., 2010). Phages can also carry harmful genes and can potentially transfer these genes to the bacteria, increasing virulence (Skurnik and Strauch, 2006). As a result, there has been interest in the use of phage proteins such as endolysins (Roach and Donovan, 2015) as tools for the specific targeting of bacteria and the exploitation of phage receptor binding proteins for use in diagnostics and engineered phage-derived killing machines (Singh et al., 2012; Simpson et al., 2015). Unexpectedly, Waseh et al. (2010) have demonstrated that the P22 phage tailspike protein alone is effective in controlling *Salmonella* colonization and spread in chickens, presumably through its binding capability. These tailspike proteins are highly stable homotrimers that form the short tail of the bacteriophage and bind to the O-antigenic repeating units on the outer membrane lipopolysaccharide (Baxa et al., 1996). The tailspike protein Gp9 from the P22 bacteriophage can recognize several serovars of *Salmonella* including *S.* Typhimurium, *S*. Paratyphi A, and *S*. Enteritidis. A shortened version of Gp9 has been shown to agglutinate *S.* Typhimurium, inhibit bacterial motility and reduce colonization in the chicken gut (Waseh et al., 2010). Therefore, this protein has the potential to act as an effective pre-slaughter feed additive to reduce *Salmonella* contamination in chickens.

Plant bioreactors have been growing in acceptance as feasible production platforms for therapeutic proteins, as they are highly scalable and can be established with little upfront cost (Fischer et al., 2012). Protein drugs expressed in plant tissue are thought to be protected from digestive enzymes by the plant cell wall (Kwon and Daniell, 2015), and are especially useful for veterinary applications where regulations allow administration of unpurified or partially purified extracts (MacDonald et al., 2015). For example, leaf tissue can be harvested, lyophilized, and orally administered in capsules or suspended in a slurry removing costs associated with protein purification, administration, and cold-storage (Kolotilin et al., 2014). As higher eukaryotic organisms, plants can introduce post-translational modifications required for complex recombinant proteins. Despite these benefits, recombinant protein yield remains a major factor limiting the widespread adoption of plant bioreactors for commercial protein production. Consequently, several approaches are currently being used to increase protein accumulation in plants. Proteins can be targeted to different subcellular compartments such as the ER, the chloroplasts, and the apoplast using signal and transit peptides (Conley et al., 2009b). This is because each subcellular compartment has a unique biochemical environment, protease content, and physical size which influence protein accumulation levels (Streatfield, 2007; Pillay et al., 2014). Additionally, peptide tags can be fused to recombinant protein to increase accumulation. For example, fusion tags such as ELPs and HFBI can increase recombinant protein accumulation levels, and have also been used to purify proteins from plant extracts (Conley et al., 2011).

The goal of this project was to transiently produce the truncated version of Gp9 in *Nicotiana benthamiana* by targeting the protein to the chloroplasts, to the ER, or to the ER fused with an ELP or HFBI tag. The activity of plant-produced Gp9 was then tested by examining its ability to bind to *S.* Typhimurium. Lastly, plant tissue containing Gp9 was orally administered to chickens inoculated with *S.* Typhimurium to determine if this plant produced therapeutic has the potential to limit *Salmonella* colonization.

## Materials and Methods

### Gene Cloning

The truncated version (encoding amino acids 109–666) of the endorhamnosidase mutant of *gp9* (as described in Waseh et al., 2010) was codon-optimized for plant expression and synthesized by Biobasic Inc. (Markham, ON, Canada). *Gp9* was recombined into the previously constructed pCaMGate expression vectors using the LR reaction of Gateway^®^ technology (Thermo Fischer Scientific, Waltham, MA, USA), courtesy of Dr. Andrew Conley of Agriculture and Agri-Food Canada, London Ontario. Recombinant pCAMGate vectors were transformed in *Escherichia coli* XL1-Blue using the Gene Pulser II system (Bio-Rad Laboratories Inc., Hercules, CA, USA) and PCR screening using gene-specific primers (Forward primer: CGTTAGGTGTAGGTTTTGGTATGGATGGT, Reverse primer: CCGGCAACAGGATTCAATCTTAA) was conducted to screen for positive transformants containing the correct insert. Plasmid DNA was isolated from positive colonies and transformed into electro-competent *Agrobacterium tumefaciens* EHA105 cells. Electroporated *A. tumefaciens* cells were spread on yeast extract broth (YEB) plates containing 50 μg/ml kanamycin and 10 μg/ml rifampicin and incubated for 2 days at 28°C.

### Transient Expression in *N. benthamiana* Plants

Suspensions of *A. tumefaciens* carrying *Gp9* or *A. tumefaciens* carrying the post-transcriptional gene silencing suppressor *p19* from Cymbidium ringspot virus (Silhavy et al., 2002), were incubated overnight at 28°C with shaking at 250 rpm until an optical density at 600 nm (OD600) of 0.5–1.0 was reached. Cultures were then centrifuged at 6000 × *g* for 30 min and resuspended to an OD600 of 1.0 in Gamborg’s solution containing 3.2 g/l Gamborg’s B5 with vitamins, 20 g/l sucrose, 10 mM MES (pH 5.6), and 200 μM acetosyringone. Cultures were then incubated at room temperature with gentle agitation for 1 h. An equal volume of *A. tumefaciens* culture containing *Gp9* was combined with *A. tumefaciens* culture carrying *p19* and Gamborg’s solution to give a total *A. tumefaciens* OD600 of approximately 0.67. These suspensions were used to infiltrate 7–8 week-old *N. benthamiana* plants grown in a growth room under 16 h light/8 h dark conditions at 21–22°C with 55% humidity, and receiving roughly 100 μmol/photons m^-2^s^-1^ of light. A 3 ml syringe was used to infiltrate the *A. tumefaciens* suspensions through the stomata of the abaxial leaf epidermis of *N. benthamiana*. After infiltration, plants were returned to the growth chamber for up to 6 days.

### Tissue Collection and Protein Extraction

Four biological replicates were used in all experiments and consisted of four plants sampled as follows: two leaf disks/leaf (7 mm diameter) were collected from three infiltrated leaves of each plant and pooled. Tissue was flash frozen in liquid nitrogen and stored at -80°C until use. For protein extraction, tissue was homogenized twice in 30 s pulses using a TissueLyser (Qiagen, Venlo, Netherlands) and TSP were extracted in 200 μl of plant extraction buffer (PEB) containing 1X phosphate-buffered saline (PBS), 0.1% (v/v) Tween-20, 2% (w/v) polyvinylpolypyrrolidone (PVPP), 100 mM ascorbic acid, 1 mM ethylenediaminetetraccetic acid (EDTA), 1 mM of phenylmethanesulfonylfluoride (PMSF) and 1 μg/ml leupeptin. TSP concentration for each sample was determined using the Bradford assay (Bradford, 1976). For electrophoresis under non-reducing conditions, protein samples were stored in a 5% (w/v) SDS, 250 mg/ml glycerol, 0.1 mg/ml bromophenol blue, 0.16 M Tris/HCl sample buffer (Seckler et al., 1989) to better visualize protein trimerization. Samples were frozen at -80°C until use.

### Western Blotting and Gel Staining

Pooled sample extracts and individual replicates were immunodetected against a standard curve of known amounts of purified Gp9-ELP to accurately quantify protein accumulation levels. Samples were either boiled for ten minutes or not boiled and loaded onto Bio-Rad Mini-Protean^®^ TGX^TM^ Precast 4–20% (w/v) polyacrylamide gradient gels. Separated proteins were transferred to polyvinylidene difluoride (PVDF) membranes and blocked overnight in a 5% (w/v) skim milk powder in TBS-T (Tris-buffered saline-Tween 20) blocking solution. Membranes were incubated with a 1:5000 dilution of mouse anti-c-Myc antibody (Genscript, A00864, Piscataway, NJ, USA) or a 1:8000 dilution of rabbit polyclonal anti-Gp9 antibody (Kropinski et al., 2011). Membranes were washed and incubated with a 1:5000 dilution of goat anti-mouse or anti-rabbit secondary antibody conjugated with horseradish peroxidase (HRP), and visualized with the GE Healthcare Life Sciences (Little Chalfont, UK) ECL Prime Western Blotting Detection Reagent. Recombinant protein was quantified by image densitometry using Totallab TL100 software (Non-linear Dynamics, Durham, NC, USA). For staining of separated proteins, gels were washed for 5 min in water and stained for 1 h with GelCode^TM^ Blue stain reagent (Thermo Fischer Scientific, Waltham, MA, USA) at room temperature. Gels were then destained using three 5-min washes with water and imaged.

### Protein Purification

Gp9-ELP and Gp9-HFBI proteins were purified using a c-Myc tag purification kit from MBL International Corporation MBL (3305, Woburn, MA, USA) according to the manufacturer’s instructions. Purified protein was stored at -80°C until use. The *E. coli* produced His_6_-Gp9 was purified as described by Waseh et al. (2010).

### Gp9 and Gp9-ELP Adherence to *S.* Typhimurium

*Salmonella enterica* serovar Typhimurium (ATCC19585) was purchased from the American Type Culture Collection (Manassas, VA, USA) and grown under aerobic conditions at 37°C on Lysogeny Broth (LB) agar plates. The strain used in the adherence assay was transformed with the pWM1007 plasmid (Miller et al., 2000) which expresses the green fluorescence protein (GFP) and grown on LB supplemented with 25 μg/ml kanamycin.

Five hundred nanograms of the *E. coli* produced His_6_-Gp9, BSA, or plant-produced Gp9-ELP were spotted onto hole punch sized pieces of nitrocellulose membranes and then blocked for 1 h in 5% (w/v) skim milk in PBS with 0.05% (v/v) Tween (PBS-T). The membranes were then probed with 10^8^ cfu/ml of GFP-expressing *S.* Typhimurium in 5% skim milk PBS-T. The disks were washed three times for 5 min in PBS-T and were then placed onto LB agar plates with 25 μg/ml kanamycin and allowed to grow at room temperature overnight, followed by growth at 37°C for 8 h. The disks were imaged with a FujiFilm (Tokyo, Japan) FLA-5000 system using the 473 nm laser at 400V for excitation and LPB (Y510) filter for emission. Fluorescence intensity was measured using the MultiGauge version 3.0 software.

### Animal Studies

Animal studies were carried out in accordance with the protocol approved by the Animal Care and Use Committee at the University of Alberta following the procedure described by Waseh et al. (2010). Each group contained 5–8 SPF leghorn chickens (Poultry Research Facility, University of Alberta) that were provided with feed and water *ad libitum* and were randomly tested for the presence of *Salmonella* on the day of hatching by plating cloacal swabs onto selective Oxoid Brilliance *Salmonella* agar (Oxoid, ON, Canada). In all cases no *Salmonella* colonies were observed after 24 h of incubation at 37°C. Chickens were orally gavaged with 300 μL PBS containing 10^7^ colony forming units (CFUs) of *S*. Typhimurium the next day and then gavaged with 35 mg lyophilized and powdered leaves resuspended in 300 μl of PBS at 1, 18, and 42 h post-infection. The chickens were culled at 47 h post-infection and the collected cecal contents were serially diluted and plated onto Oxoid Brilliance *Salmonella* agar. *Salmonella* CFU were counted after the agar plates were incubated for 24 h at 37°C.

### Statistics

Minitab^®^ 17 statistical software (Minitab Ltd., Coventry, UK) was used to perform statistical analysis on the Gp9 accumulation data. A one-way analysis of variance (ANOVA) was performed with a Tukey test on the mean Gp9 accumulation levels for each day of the time course. *P* < 0.05 was considered significant. A two-tailed Student’s *t*-test was performed on the data from the chicken experiment. *P* < 0.05 was once again considered significant.

## Results

### Gp9 Transient Expression in *N. benthamiana*

**FIGURE 1:**
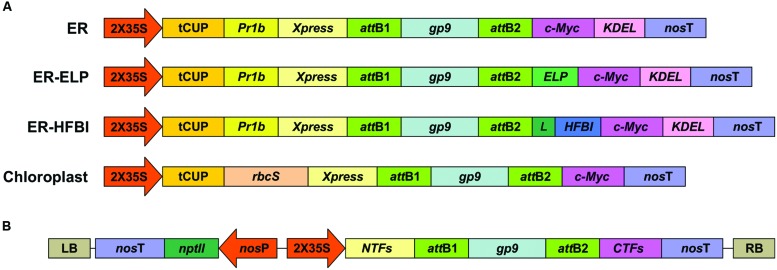
**Expression cassettes for Gp9 transient expression in *N. benthamiana*.** **(A)** A schematic representing the gene constructs targeting the ER or the chloroplasts. Two of the cassettes targeting the ER also contain sequences coding for an ELP or a HFBI tag with a linker (L). Expression and targeting elements incorporated into the expression vectors are: the double-enhanced 35S cauliflower mosaic virus promoter (2X35S); tCUP translational enhancer from tobacco; tobacco Pathogenesis-Related-1b signal peptide (PR1b) and the KDEL ER retrieval tetrapeptide; nopaline synthase terminator (*nos*T); and *Xpress* and *c-Myc* tags for detection and purification. A sequence coding for the RuBisCo small subunit transit peptide (*rbcS*) was used for targeting to the chloroplasts. **(B)** A zoomed-out schematic of the constructs in the T-DNA sequence. LB and RB are the left and right borders of the T-DNA sequence. *nptII* codes for neomycin phosphotransferase conferring resistance to kanamycin, driven by a nopaline synthase promoter (*nos*P) and terminator (*nos*T). Schematic represents the same constructs in **(A)** with different N-terminal fusions (*NTFs*) and C-terminal fusions (*CTFs*). Schematics are not to scale.

**FIGURE 2:**
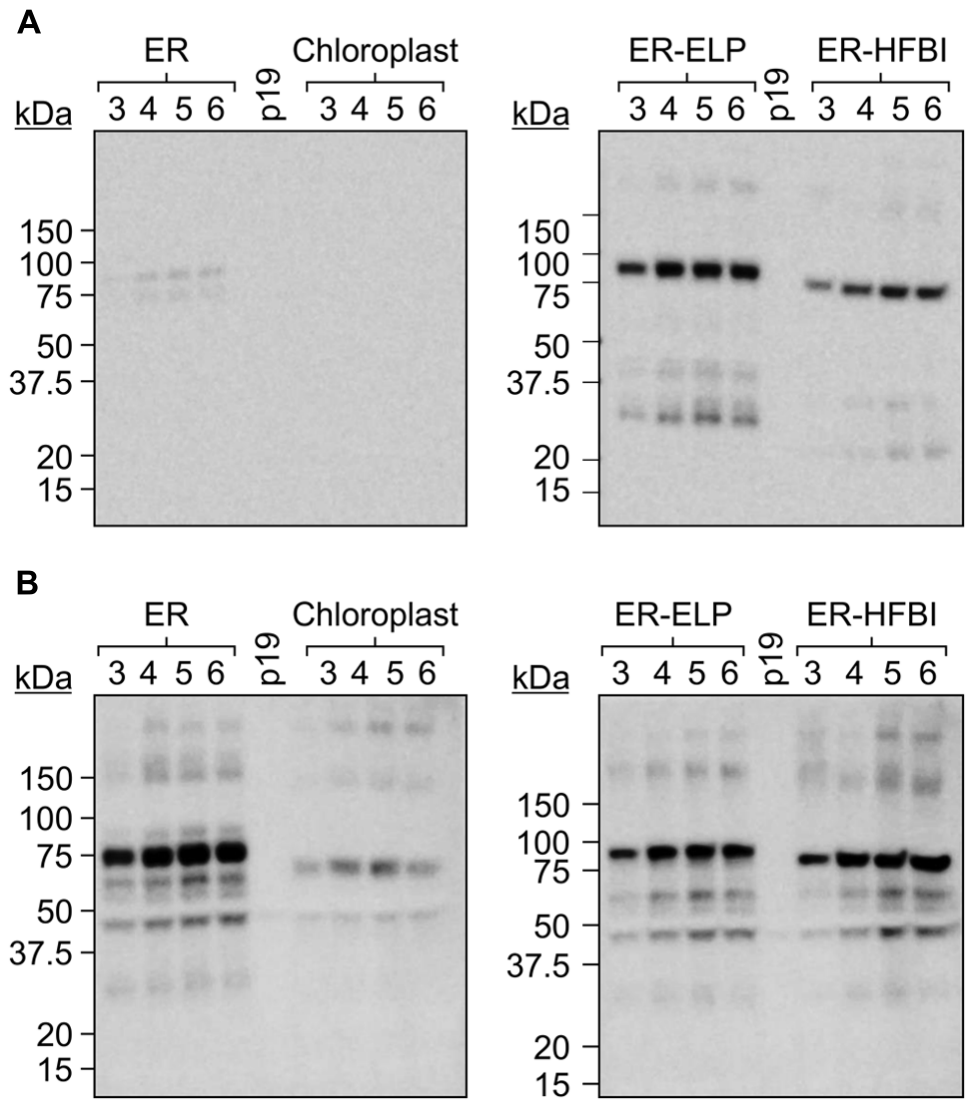
**A time course of Gp9 accumulation levels in *Nicotiana benthamiana*.** **(A)** Immunoblots of TSP extracted from *N. benthamiana* leaf tissue infiltrated with *gp9* constructs targeting the ER, the chloroplasts, or the ER fused to an ELP tag (ER-ELP) or fused to a hydrophobin tag (ER-HFBI). Four plants were infiltrated with each construct. Tissue was sampled from infiltrated leaves from 3 to 6 dpi. Equal volumes of TSP from each of the four replicates were pooled, and 20 μg was loaded per well. Immunoblots were probed with **(A)** an anti-c-Myc antibody or **(B)** an anti-Gp9 antibody. p19: TSP from plants infiltrated with p19 serving as a negative control.

The *Gp9* tailspike gene was cloned into pCaMGate expression vectors (Pereira et al., 2014) targeting the ER, the ER fused with an ELP tag (ER-ELP), the ER fused with a HFBI tag (ER-HFBI), or the chloroplasts (**Figure 1**). Constructs were then agroinfiltrated along with the gene silencing suppressor, p19 (Silhavy et al., 2002), into the leaves of *N. benthamiana* plants. Plants were monitored over the course of 6 day(s) post-infiltration (dpi). Young, upper leaves infiltrated with the ER-targeted constructs turned a slight yellow–green color but otherwise the phenotypes remained unchanged. Leaf tissue samples were collected from 3 to 6 dpi and analyzed for Gp9 accumulation via Western blot using a c-Myc antibody specific for the C-terminal Myc peptide found in all constructs (**Figure 2A**). Gp9 targeted to the ER was faintly visible, running higher than predicted (**Table 1**), and no bands were visible for the chloroplast-targeted Gp9. Conversely, Gp9-ELP and Gp9-HFBI were readily detected, and faint bands were observed for both the ELP and HFBI fused constructs migrating above 150 kDa which could represent potential trimers. Smaller bands ranging in size between 20 and 50 kDa were also observed in the Gp9-ELP and Gp9-HFBI samples which most likely correspond to Gp9 degradation products since they are absent from the p19 negative control lane (**Figure 2A**). Interestingly, when immunoblots were probed with an anti-Gp9- antibody, a strong band slightly under 75 kDa was observed for ER-targeted Gp9, and a somewhat fainter band was seen in the chloroplast-targeted Gp9 (**Figure 2B**). Bands potentially representing dimers and trimers were also observed for all four proteins, as well as smaller bands that may represent Gp9 degradation products. It is interesting that the smaller bands observed in the Gp9-ELP and Gp9-HFBI are of a different size when detected with the two antibodies. The protein band migrating between 37.5 and 50 kDa may be a plant protein as it appears faintly in the p19 lane, while the band running between 50 and 75 kDa may represent a degradation product of Gp9. This band is present in all samples, and it may represent the N-terminal portion of the protein detected with the Gp9-specific antibody, while the smaller band running between 20 and 37 kDa on the blot detected with the c-Myc antibody might represent the C-terminal portion of the protein. The low abundance of Gp9 on blots probed with the c-Myc antibody also imply that the c-Myc tag is cleaved off Gp9, or is inaccessible to the antibody.

**Table 1:** Predicted molecular weights of Gp9 constructs.

Construct	Predicted full-length size	Predicted size without c-Myc tag	Predicted size without C-terminal fusions
ER	67	65.33	65.33
ER-ELP	78.47	76.8	65.33
ER-HFBI	74.78	73.63	65.33
Chloroplast	66.52	65.33	65.33


*Values are in kiloDaltons (kDa).*

### Gp9 Accumulation in *N. benthamiana*

**FIGURE 3:**
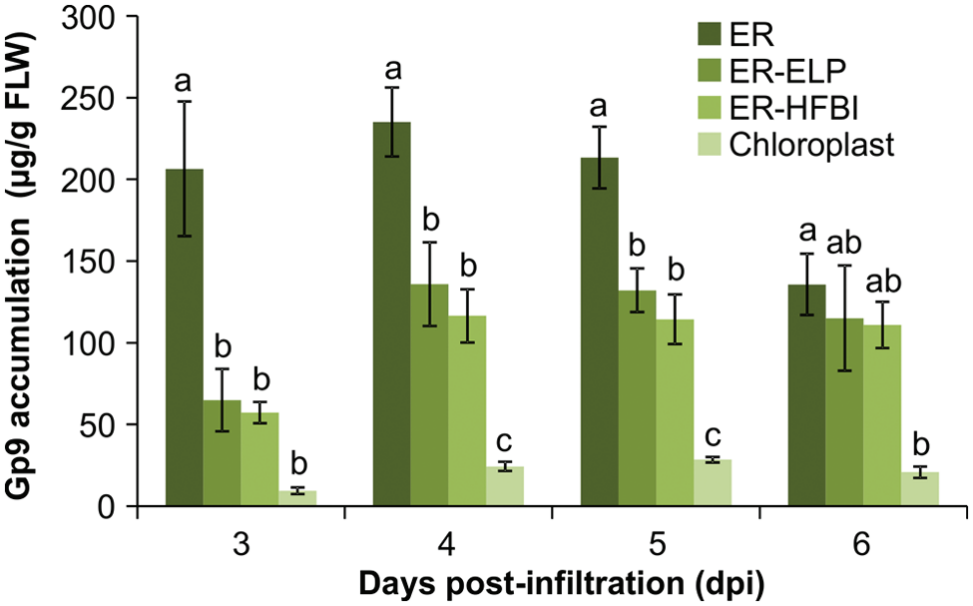
**Quantification of Gp9 accumulation in *N. benthamiana* over 3, 4, 5, and 6 dpi.** Quantification was performed on TSP extracts from infiltrated *N. benthamiana* tissue using a standard curve of known amounts of purified Gp9-ELP. Gp9 was targeted to the ER, the chloroplasts, or the ER fused to an ELP tag (ER-ELP) or fused to a hydrophobin tag (ER-HFBI). Immunoblots were probed with an anti-Gp9 antibody. Accumulation levels of Gp9 are shown in μg per g of FLW. Error bars represent the standard error of the mean value of four biological replicates. Treatments which do not share a letter are significantly different (*P* < 0.05) as determined by a one way ANOVA and the Tukey test.

Since more protein was detected using the anti-Gp9-antibody, this antibody was used to accurately quantify Gp9 accumulation via densitometry analysis. For the purpose of protein quantitation, only bands representing the full length Gp9 monomer were quantified. Immunoblots using individual replicates revealed that Gp9 accumulation increases from the 3rd dpi, peaks on day 4 or 5, and subsequently decreases (**Figure 3**). ER-targeted Gp9 accumulated in significantly higher amounts than the other proteins reaching on average of 1.64 ± 0.09% of TSP on day 5. When recombinant protein accumulation is calculated in terms of micrograms of Gp9 per gram of FLW, Gp9 accumulates to an average of 235.01 ± 21.12 μg/g of FLW on day 4 (**Figure 3B**). The presence of either the ELP or HFBI tag appears to significantly decrease Gp9 accumulation on 3–5 dpi (*P* < 0.05). Gp9-ELP accumulates on average to 0.98 ± 0.05% of TSP on day 5 or 135.86 ± 25.56 μg/g of FLW on day 4, roughly 0.6 times or 1.7 times less, respectively, than when unfused. The presence of the HFBI tag caused Gp9 to accumulate in even lower amounts on average to 0.79 ± 0.09% of TSP (0.48 times less) or 116.49 ± 16.29 μg/g of FLW (two times less) on day 4. Gp9 accumulated significantly less when targeted to the chloroplasts compared to ER-targeted proteins on days 4 and 5 (*P* < 0.05). Accumulation reached an average of 0.21 ± 0.01% of TSP or 28.45 ± 1.65 μg/g of FLW on the 5th dpi.

### Gp9-ELP Purification and Characterization

**FIGURE 4:**
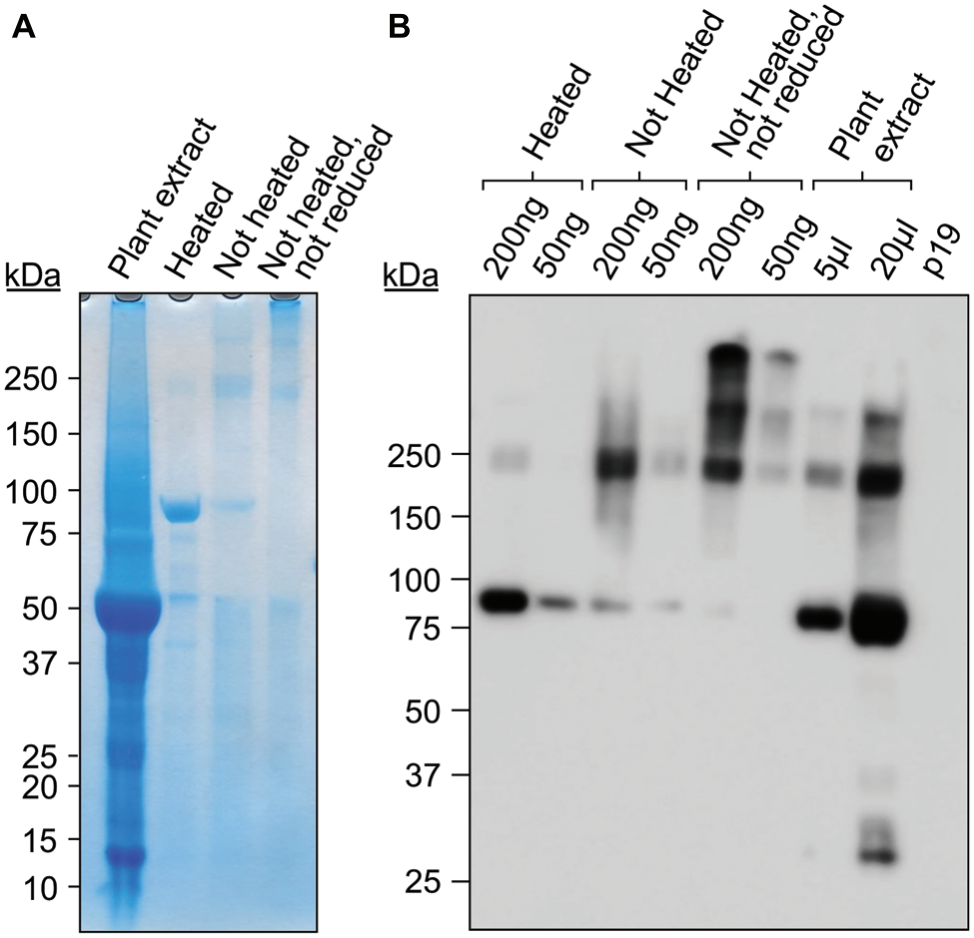
**Purified Gp9-ELP is found in higher molecular weight protein complexes.** Purified Gp9-ELP was electrophoresed under the following conditions: in sample buffer containing DTT and heat-denatured by boiling for 10 min, in sample buffer containing DTT with no heat denaturation, or in sample buffer without DTT and no heat denaturation. The plant extract containing Gp9-ELP was also loaded as a positive control in sample buffer containing DTT and heat-denatured by boiling for 10 min. Twenty microliters of extract from plants infiltrated with p19 were used as a negative control. Gels were either coomassie stained **(A)** or immunodetected **(B)** using an anti-c-Myc antibody.

Gp9 polypeptides are found in monomeric, dimeric, and trimeric intermediates before forming the stable, native trimer (Benton et al., 2002). The trimeric intermediate species or protrimer consists of associated subunits which have not completely folded forming a transient, less-stable precursor to the trimer (Goldenberg and King, 1982). We successfully purified Gp9-ELP using a c-Myc-tag purification kit (**Figure 4A**) and decided to investigate if purified Gp9-ELP is present in either of these states by avoiding complete denaturation (unheated sample) and by avoiding reducing agents such as dithiothreitol (DTT) in the sample buffer to keep the disulfide bonds oxidized (unheated, no DTT). While most of the Gp9-ELP was found in the monomeric form when it was reduced and denatured, there was very little monomer present when heat denaturation was omitted. Instead, an intense band was observed below 250 kDa, which corresponds to the expected size for the 215 kDa trimer (**Figures 4A,B**). Higher banding was observed when samples were electrophoresed under non-reducing conditions (**Figure 4B**). Gp9 contains eight cysteine residues and has disulfide bonds while existing as a protrimer, despite the fact that all are reduced during conversion to the native trimeric state (Robinson and King, 1997). Consequently, bands running above 250 kDa could represent the protrimer intermediate running slower than the trimer. Generally, an oxidizing environment is needed for trimer folding due to the presence of the disulfide bonds in the protrimer, yet increasing concentrations of DTT increases the conversion of protrimer to trimer (Robinson and King, 1997). Even larger bands were visualized and could represent higher-order multimers or aggregates (Speed et al., 1997).

Our results suggest that Gp9 is present in the stable trimeric form when purified and is expected to remain functional to some degree.

### Gp9-ELP Binding to *S.* Typhimurium

**FIGURE 5:**
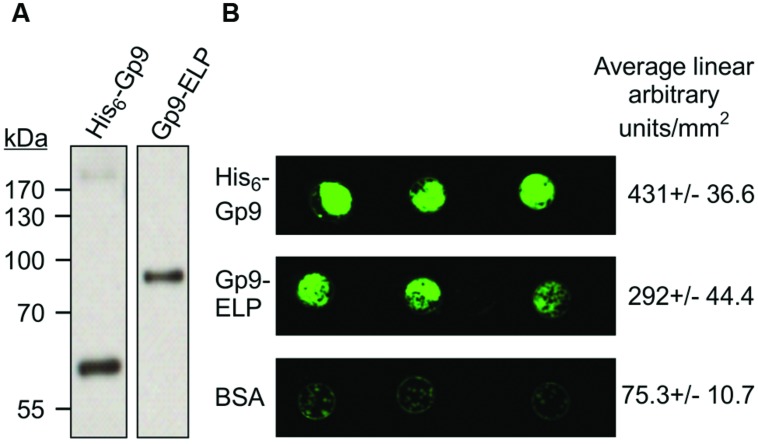
***S.* Typhimurium binding activity to plant produced Gp9-ELP.** **(A)** The *Escherichia coli* produced His_6_-Gp9 (62 kDa) and the plant-produced Gp9-ELP (82 kDa) both react with anti-Gp9 antibodies. **(B)** Either 500 ng of His_6_-Gp9, BSA, or Gp9-ELP protein were spotted onto nitrocellulose and then probed with 10^8^ cfu/ml GFP-expressing *S.* Typhimurium and allowed to grow overnight. Two biological replicates were done, each with three technical replicates in each. One representative image and densitometry analysis is shown.

To determine if the plant produced Gp9 can bind to *S.* Typhimurium, 500 ng of purified Gp9-ELP was spotted onto a nitrocellulose membrane, blocked and then probed for 1 h with 10^8^ cfu/ml of GFP expressing *S.* Typhimurium. BSA and *E. coli*-produced Gp9 were used as negative and positive controls, respectively. Gp9-ELP was able to bind *S.* Typhimurium at similar levels as the *E. coli-*produced Gp9 as measured by fluorescence (**Figure 5**) suggesting that the plant-produced protein is functional.

### The Effect of Oral Administration of Gp9-ELP on *Salmonella* Colonization

**FIGURE 6:**
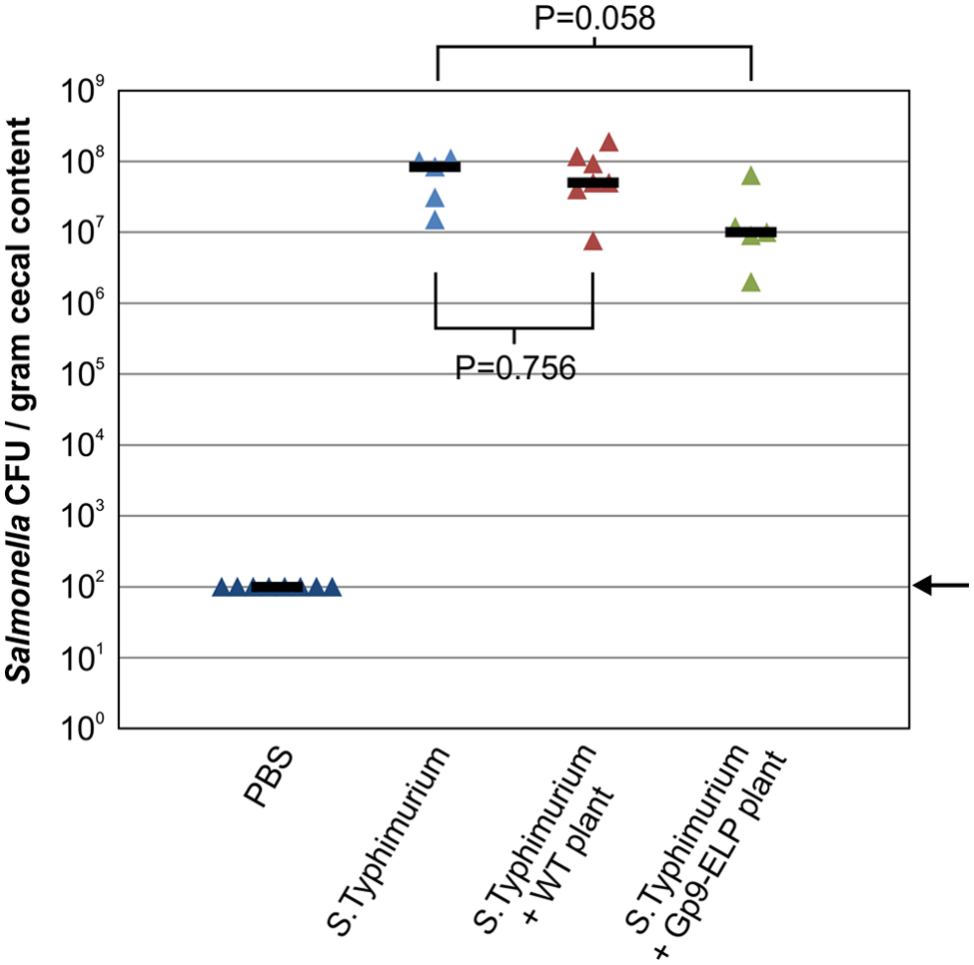
**Treatment of *S*. Typhimurium infected chickens with Gp9-ELP.** SPF leghorn chickens were orally gavaged with PBS or *S*. Typhimurium as indicated. One hour later (and at 18 and 42 h), two of the groups were orally gavaged with *N. benthamiana* extracts with or without Gp9-ELP, while the control groups received PBS. Chickens were fed 7.7 μg of Gp9-ELP per dose. *Salmonella* colonization levels of the chicken ceca were determined 47 h post-infection. The median is shown for each group and *P*-values were determined for the plant-fed groups in comparison to the *S*. Typhimurium group. The arrow represents the detection limit of the experiment.

Due to the demonstrated binding activity of Gp9-ELP, we investigated the effects of orally administering plant tissue containing Gp9-ELP on *Salmonella* colonization in chicks. The chicks were orally gavaged with 35 mg of resuspended plant tissue containing 7.7 μg Gp9-ELP 1, 18 and 42 h after gavaging with *S*. Typhimurium. Chicks were culled 47 h after bacterial infection and the *Salmonella* CFU in the cecal contents were enumerated (**Figure 6**). Gp9-ELP showed approximately 1-log reduction in *S*. Typhimurium colonization compared to the untreated control birds (*P* = 0.058). These results are promising considering plant leaves were fed directly to the birds without further purification, and each dose contained much less Gp9-ELP compared with the Waseh et al. (2010) study where a dose consisted of 30 μg of purified Gp9.

## Discussion

This study demonstrated that the receptor binding domain of the P22 bacteriophage tailspike protein, Gp9, could be transiently expressed in *N. benthamiana* at reasonable levels and is able to bind to *S.* Typhimurium. The results presented here suggest that the correct choice of antibody for protein detection is essential to accurately quantify recombinant protein accumulation. When probing with an antibody specific to the C-terminal Myc tag, Gp9 appeared to accumulate to the highest levels when fused to an ELP or HFBI tag, supporting the hypothesis of this study that these fusion tags would increase accumulation levels as previously reported (Patel et al., 2007; Conley et al., 2009a; Joensuu et al., 2010). These results were expected as ELP and HFBI tags promote the formation and distribution of protein bodies (PBs) which are thought to protect recombinant protein from hydrolysis and protease cleavage (Conley et al., 2011; Saberianfar et al., 2015).

However, probing with a Gp9-specific antibody gave contrasting results. When the Gp9-specific antibody was used for immunodetection, unfused ER-targeted Gp9 displayed about 50% more accumulation than the ELP and HFBI fusions. This result was unexpected, and suggests that Gp9 is stable in *N. benthamiana* and the additional ELP or HFBI tags do not increase protein stability further. Indeed, ELP tags have negligible effects on already high accumulating proteins such as GFP (Conley et al., 2009a). It appears that the addition of an ELP or HFBI tag actually decreases Gp9 accumulation when targeted for retrieval to the ER of *N. benthamiana* and this should be investigated in future studies. These results also imply that the c-Myc tag is partially cleaved or inaccessible causing us to underestimate Gp9 accumulation levels when detecting with an anti-c-Myc tag antibody. It is possible that the ELP and HFBI tags protect the c-Myc tag from cleavage, possibly through protein body formation, and therefore the fusion constructs were able to be detected with the c-Myc antibody. However, when probing with the Gp9 antibody the ER and chloroplast constructs produce a slightly higher banding pattern than expected if the c-Myc is indeed cleaved off (**Figure 2**; **Table 1**). It can be postulated then that these proteins may fold into a conformation that makes the c-Myc tag inaccessible. Regardless, as many recombinant proteins are designed to have tags for easy detection, these results are a cautionary note on the limits of short tags, and that perhaps both N- and C-terminal tags might be used to improve chances of accurate quantitation, while protein-specific antibodies are most probably the best option if available. Our constructs also contain an N-terminal Xpress tag (**Figure 1**), however, commercially available antibodies have not detected any recombinant protein in crude extracts (unpublished).

Gp9 also accumulated in the chloroplasts, but to a lower level than when targeted to the secretory pathway. Generally, chloroplast targeting is a promising strategy for increasing recombinant protein accumulation (Hyunjong et al., 2006). Nevertheless, some proteins do not accumulate well when targeted to these organelles. For example, one study described lower accumulation levels of zeolin, a chimeric storage protein, when targeted to the chloroplasts compared to the ER. A visualized degradation pattern of zeolin provided evidence for protein degradation in the chloroplasts (Bellucci et al., 2007). On the other hand, Oey et al. (2009) were able to express a phage lysin protein in tobacco chloroplasts with accumulation levels reaching 70% of TSP. This protein was produced by transforming the plastid genome and the gene was codon-optimized for plastid expression. The phage lytic protein was shown to be extremely stable in the plastids, which is understandable as phages have evolved substantial resistance to bacterial proteases. By analogy, the Gp9 should be stable in the plastids, and therefore it is possible that the protein is unable to efficiently translocate into the chloroplasts. Therefore, future work could focus on transforming the plastid genome of *N. tabacum* for high level, stable expression of Gp9 without having to rely on chloroplast protein import.

Like the full length Gp9, shortened versions lacking the amino acid N-terminus domain maintain the stability and enzymatic activity of the full length parent proteins (Miller et al., 1998). To the best of our knowledge, this is the first study to transiently express this truncated protein in plants. Previously, truncated tailspikes have been expressed in *E. coli*, isolated and shown to reduce *Salmonella* colonization in the chicken GI tract. It is believed that the tailspikes bind to *Salmonella* and retard its motility and binding capability (Waseh et al., 2010). Consequently, plant-produced Gp9 has the potential to serve as a therapeutic to control *Salmonella.* These proteins can potentially be added to chicken or other livestock feed, reducing contamination and reducing infections in humans. While the plant produced Gp9 did not yield statistically significant results in the chicken experiment, the results are indeed promising. We purified Gp9-ELP using a Myc column to show that Gp9-ELP exists as a trimer and remains active by binding to *S.* Typhimurium. However, it is still possible that the ELP tag could alter Gp9 conformation *in vivo*, influencing activity and future work could focus on unfused Gp9. *In vitro*, ELPs have been reported to reduce protein activity compared to non-fused proteins (Shamji et al., 2007; Kaldis et al., 2013). It is possible that the physiological temperature of chickens could influence the solubility of Gp9-ELP. This is because ELPs undergo a reversible phase transition from soluble to insoluble aggregates when heated past a certain transition temperature. However, this transition temperature can be manipulated by the altering the number and/or the residue composition of the peptide repeats in order to adjust the solubility of Gp9-ELP, thus allowing for better therapeutic efficacy at physiological temperatures (Hassouneh et al., 2012). Furthermore, it is likely that the 1 log reduction in *Salmonella* colonization observed in this study compared to the 2 log reduction in the Waseh et al. (2010) study is due to the lower Gp9 dose administered to the birds (30 μg of purified His_6_-Gp9 versus 7.7 μg Gp9-ELP in 35 mg of lyophilized plant tissue per dose) as well as the fact that the Gp9-ELP protein is contained within plant cells and thus the chicken’s gut proteases need to digest through the plant cell wall in order to release the protein. In the previous study, it was shown that if the first dose was delivered at 18 h instead of 1 h post-infection, the benefits of the treatment were less. In our study, the protein would be released from the plant tissue sometime after the treatment is administered, so it is possible that this delay is partially responsible for the lower reduction in infection. Future studies will compare administration of higher levels of Gp9 protein and providing the feed throughout the experiment in order to more closely resemble the natural conditions in which this protein would be used.

One of our goals in this study was to demonstrate that Gp9 could be directly administered while in minimally processed plant tissue, without having to rely on taxing purification and formulation techniques. It can be postulated that purified Gp9 may better reduce *Salmonella* colonization in chickens as previously reported (Waseh et al., 2010). While a direct comparison between purified and non-purified Gp9-ELP was not done in this study, we have shown that wild type plant tissue alone is not significantly influencing *S*. Typhiumurium colonization in chicks. Therefore, while future work should mainly focus on increasing Gp9 accumulation in *N. benthamiana*, this study serves as another successful example of engineering plant factories for the production of a functional therapeutic.

## Author Contributions

SM and DS designed the research, performed the experiments, SM wrote the manuscript. RM, CS, and MD conceived the study, participated in its design and edited the manuscript.

## Conflict of Interest Statement

The authors declare that the research was conducted in the absence of any commercial or financial relationships that could be construed as a potential conflict of interest.

## Footnotes

1 http://healthycanadians.gc.ca/alt/pdf/publications/drugs-products-medicaments-produits/antibiotic-resistance-antibiotique/antimicrobial-surveillance-antimicrobioresistance-eng.pdf

## Abbreviations

BSA: bovine serum albumin
CFU: colony-forming units
ELP: synthetic elastin-like polypeptide
ER: endoplasmic reticulum
FLW: fresh leaf weight
GI tract: gastro-intestinal tract
HFBI: hydrophobin I from *Trichoderma reesei*
*S*. Typhimurium: *Salmonella enterica* serovar Typhimurium
*S*. Paratyphi: *Salmonella enterica* serovar Paratyphi
*S*. Enteritidis: *Salmonella enterica* serovar Enteriditis
*S.* Heidelberg: *Salmonella* Heidelberg
TSP: total soluble protein(s).
